# Sensory and Volatile Profiles of Korean Commercially Distilled *Soju* Using Descriptive Analysis and HS-SPME-GC-MS

**DOI:** 10.3390/foods9091330

**Published:** 2020-09-21

**Authors:** Jung-Min Hong, Tae-Wan Kim, Seung-Joo Lee

**Affiliations:** 1 Department of Culinary and Food Service Management, Sejong University, Neungdong-ro 209, Gwangjin-gu, Seoul 05006, Korea; jmhong@sejong.ac.kr; 2 Traditional Foods Division, Korea Food Research Institute, Nongsangmyong-ro 245, Iseo-myeon, Wanju-gun, Jeollabuk-do 55365, Korea

**Keywords:** liquor, *soju*, GC-MS, SPME, volatile compounds, sensory analysis, partial least squares regression analysis

## Abstract

Volatile compositions and sensory characteristics of 11 commercially distilled *soju* samples were investigated using headspace solid-phase microextraction (HS-SPME) with gas chromatography-mass spectrometry (GC-MS) and sensory descriptive analysis. A total of 59 major volatile compounds, consisting of 32 esters, 10 alcohols, 2 acids, 5 aldehydes, 3 ketones, 1 hydrocarbon, 1 furan, 2 phenols, and 3 miscellaneous compounds, were identified. From the principal component analysis (PCA) of volatile data, MSJ made by atmospheric distillation showed a clear distinction in volatile compositions compared to that of other samples made by vacuum distillation. Based on PCA of the sensory data determined by a panel of ten judges, MSJ was associated with a large amount of longer chain esters that showed high intensities in bitter taste and yeast/*nuruk*-related flavor attributes. HYJ, LPJ, and HAJ made with rice as a raw material were associated with lower intensities of the alcohol aroma, while JRJ and OKJ aged in oak barrels were associated with fruit flavor, sweet flavor, and brandy aroma. In the partial least squares regression (PLSR) analysis to see any relationship between volatile and sensory data, longer chain esters like ethyl tetradecanoate, and ethyl hexadecanoate were highly associated with bleach aroma. In contrast, positive correlations were seen with barley aroma and yeast flavor with hexanal, nonanal, benzaldehyde, and 2-methoxy-phenol.

## 1. Introduction

*Soju* is a well-known Korean alcoholic beverage along with unrefined rice wine, *makgeolli*. *Soju* is a distilled liquor made through a pot still, traditionally, or in a modern continuous still of fermented rice mashes (or other starchy sources) that have gone through the process of simultaneous saccharification and fermentation [1].

*Soju* is classified by its distilling process into distilled *soju* made by pot still or diluted *soju* made by decreasing the alcoholic content of liquor after continuous distillation in Korea. Distilled *soju* is known to have a complex aroma and rich flavor [2,3]. Unlike distilled *soju*, diluted *soju* has an even quality with a simple taste and aroma [4]. The production methods of distilled *soju* are separated into atmospheric distillation and vacuum distillation. The atmospheric distillation is a traditional method in which the bitter taste, grain flavor, and heated flavor of final distilled spirits are increased. In the case of modern vacuum distillation, the heated flavor of distilled liquor is lessened, and a lot of volatile compounds are preserved. Thus, vacuum distillation is gradually becoming the main production method for distilled *soju*. Recently, while the market for distilled *soju* has been rapidly increasing, there has also been increasing consumer interests for distilled *soju* with complex aroma qualities.

For the analysis of volatile compounds in hard liquors like whiskey [5], Chinese baijiu [6], sherry brandy [7], and rum [8], headspace solid-phase microextraction (HS-SPME) is widely used to prevent the interference of high levels of alcohol during the extraction. With the benefit of reduced time for extraction and sensitivity using a wide range of fibers, HS-SPME methodology has become the general application for the analysis of volatiles in alcoholic beverages as well as foods in general [9,10].

The chemical compositions including the distribution of aroma compounds depend on the fermenting of raw materials, distilled conditions, and stages of maturity of the distilled liquors. Substantial qualitative and quantitative differences in volatile compounds have been identified in many different distilled spirits [11,12]. Volatile compounds in rice-distilled *soju* aged in pottery, oak, or stainless steel tanks for 18 months were isolated by headspace solid-phase microextraction (HS-SPME) [13]. The *soju* samples aged in oak showed higher concentrations of ketones, aldehydes, and miscellaneous compounds. Volatile compounds of commercial Japanese *shochu* samples made with different raw materials and distillation methods (atmospheric vs. reduced pressure) were also reported to have ethyl acetate, ethyl pentanoate, ethyl hexanoate, ethyl octanoate, iso-amyl acetate, iso-amyl alcohol, and 2-phenylethanol as their major components [14]. These ester compounds are the main aroma compounds of most alcoholic beverages including *makgeolli*, Korean rice wine [15]. To reduce the volatile compounds that had a negative sensory impact on *soju,* volatile compounds were analyzed after distillation of *soju* mash under reduced pressure using various fermentation starters [16].

Not only are there great differences in aroma compounds according to various manufacturing conditions, but there are also big differences in sensory characteristics depending on those modifications [16,17]. However, there have been few studies about quantitative sensory characteristics of commercially distilled *soju*. In the research for sensory characteristics of nine commercially distilled *soju* samples, it was reported to have various aroma characteristics such as pungent, sour, sweet, fruity, winey, yeasty, and oak, and a spectrum of tastes such as sweetness, sourness, and bitterness [18]. Volatile compounds of distilled liquor greatly affect the quality and sensory characteristics of the products. In this aspect, systematically analyzing volatile aroma compounds of Korea’s distilled *soju* and revealing their correlation is important in improving the quality and development of new products in the distilled *soju* market.

In this study, volatile compositions were analyzed by headspace solid-phase microextraction (HS-SPME) and gas chromatography-mass spectrometry (GC-MS) by selecting eleven commercially distilled *soju* samples that had differences in fermentation of raw materials and distillation methods. Through sensory descriptive analysis, sensory characteristics were determined. To clearly understand the relationship between volatile aroma compositions and sensory characteristics, a partial least squares regression analysis (PLSR) was performed.

## 2. Materials and Methods

### 2.1. Materials and Chemicals

Eleven distilled *soju* samples were purchased from different manufacturing companies. The eleven distilled *soju* samples included products that had been made by large manufacturers with a large market share, which had won awards at the Korea Liquor Contest or were locally well-known products. For distillation methods, MSJ using the traditional atmospheric distillation method and other samples using the modern vacuum distillation were selected. Sample products were selected according to various raw materials of grains such as 100% rice, barley, millet, and sorghum. In addition, distilled *soju* aged in oak casks were selected to diversify the materials used in the experiment. Detailed information about the samples is presented in Table 1. As the internal standard for GC-MS, 2-methyl-1-pentanol, alkane standards (C9-C25), and sodium chloride were purchased from Sigma-Aldrich (ST. Louis, MO, USA).

### 2.2. Volatile Compounds Analysis

#### 2.2.1. Headspace Solid-Phase Microextraction and Gas Chromatography-Mass Spectrometry Analysis

Three commercially available SPME fibers from Supelco (Bellefonte, PA, USA) were examined for this study. These were polydimethylsiloxane/divinylbenzene (PDMS/DVB; 65 μm), carboxen/polydimethylsiloxane (CAR/PDMS; 85 μm), and polydimethylsiloxane/divinylbenzene/carboxen (PDMS/DVB/CAR; 50/30 μm), which all showed different affinities to diverse compounds. The SPME fiber (50/30 μm PDMS/DVB/CAR; Supelco, PA, USA) was selected because this fiber showed a higher affinity toward a wide range of volatile compounds. The alcohol content of all distilled *soju* samples used in the experiment was adjusted to a 10% (W/V) alcohol level using distilled water. NaCl (1 g) was added to the diluted samples (5 g) in 20 mL septum-sealed glass vials with screw caps. During sampling, 100 μL of the internal standard 2-methyl-1-pentanol (100 ng/mL in distilled water) was added to 5 g of the sample. The volatile compounds of the samples were extracted and absorbed at 40 °C for 30 min after shaking the vial at 60 °C for 10 min in an autosampler (CombiPAL G6504-CTC; CTC Analytics, Zwingen, Switzerland). Volatile compounds were analyzed using on an Agilent Technologies gas chromatograph model 7890A coupled to an Agilent 5975C mass spectrometer (Agilent Technologies, Santa Clara, CA, USA) and equipped with a Stabilwax-DA bonded fused capillary column (30 m × 0.25 mm i.d., 0.25 μm film thickness, Restek, Bellefonte, PA, USA). Mass spectral ionization was set at 220 °C, and the mass spectrometer was operated in the electron ionization mode at a voltage of 70 eV. The flow rate of nitrogen on the column was 1.3 mL/min. A 0.75 mm liner was used, analysis was performed in the splitless mode, and injector and detector temperatures were both 220 °C. The oven temperature was held at 40 °C for 5 min, programmed to increase by 5 °C/min up to 185 °C, and then was held for 20 min isothermally.

#### 2.2.2. Identification and Quantitation of Volatile Compounds

Volatile compounds were positively identified by comparing Kovats retention indices [19] and the MS fragmentation patterns with those of reference compounds or with mass spectra in the Wiley 275 mass spectral database (Hewlett-Packard, Palo Alto, CA, USA). The Kovats Indices (KI) of unknown compounds were determined via sample injection with a homologous series of alkanes (C9–C25). The GC/MS conditions were the same as described in Section 2.2.1. To quantify the volatiles, the samples were run in duplicate, and the integrated areas based on the total ion chromatograms were normalized to the areas of the internal standard and averaged. The relative concentrations of volatile compounds were determined by comparison with the concentration of the internal standard (2-methyl-1-pentanol), assuming a response factor of 1.

### 2.3. Sensory Descriptive Analysis

Sensory evaluation of the 11 distilled *soju* samples was conducted by 10 judges (3 males and 7 females) drawn from Sejong University, Seoul, Korea. Six 2 h training sessions were held for descriptor development, definitions, and panel training. To prevent strong alcohol interference during the sensory tasting, the alcohol level of the samples was adjusted to 20% W/V with distilled water. This practice is widely implemented in the whiskey industry.

In the preliminary experiment, the panel could describe aromas without difficulty and all the samples were evaluated in the same way with the same alcohol level. A total of 22 attributes were generated to characterize the sensory properties of the samples (Table 2). Standards used to define these aromas and taste descriptors were included during the training and formal sessions. The samples per session were evaluated, in duplicate, and a total of eight sessions were conducted. The presentation order of each sample was randomized for each session. The panel members were presented with 50 mL aliquots in clear plastic glasses marked with 3-digit numbers at 18–21 °C and covered with Petri dishes. The judges scored each attribute on a scale of 0–9, where 9 was the highest intensity, and 0 was none. Water and white bread were provided to the panelists for rinsing their palates between samples. All evaluations were made in sensory booths at room temperature.

### 2.4. Statistical Analysis

All statistical analyses of the GC and sensory data were performed using SAS ver.6.12 (SAS Institute, Cary, NC, USA) or XLSTAT ver. 2007.1 (Addinsoft, New York, NY, USA). The descriptive data set was initially analyzed for significant overall product effects by employing a 3-way mixed model ANOVA (judges, samples, and replications), with all 2-way interactions with judges treated as random. Individual product differences were identified by Fisher’s least-significant difference (LSD) test. The mean sensory intensities were used to perform principal component analysis (PCA) using the covariance matrix with no rotation on XLSTAT. PCA was also performed on the mean concentrations of 59 volatiles in 11 samples using the correlation matrix. In addition, correlations between mean attribute ratings and volatile concentrations were calculated. To explore the relationship between this sensory data and the volatiles for these 11 samples, a partial least squares regression analysis (PLSR) was conducted using XLSTAT ver. 2007.1 (Addinsoft, New York, NY, USA). Twelve sensory aroma attributes and 59 volatile compounds found in more than three samples were used for PLSR modeling. In PLSR, the volatiles were standardized (mean/standard deviation), while the aroma intensity ratings were assigned a weighting of 1 (unstandardized). In the PLSR model, the GC data were treated as the independent variables (X matrix), with the sensory data used as the dependent variables (Y matrix).

## 3. Results and Discussion

### 3.1. Compositions of Volatile Compounds

Table 3 lists the identified volatile components in each of the 11 samples by their chemical classes, relative concentrations, and KIs on the Stabilwax column, respectively. Initially, a total of 159 volatile compounds were detected. Among them, 59 volatile compounds detected in more than three samples were identified, including 32 esters, 10 alcohols, 2 acids, 5 aldehydes, 3 ketones, 1 hydrocarbon, 1 furan, 2 phenols, and 3 miscellaneous compounds. Esters and alcohols were the largest groups among the quantified volatiles and 10 volatile compounds were found in all samples. About 70% of the total volatiles in the 11 samples were made up of isoamyl acetate, ethyl octanoate, ethyl hexanoate, ethyl decanoate, ethyl dodecanoate, isoamyl alcohol, and 2-phenyl ethanol.

A total of 32 esters were detected and were among one of the largest classes of detected compounds. All the detected esters were previously found in various types of alcoholic beverages [20,21]. Isoamyl acetate, ethyl octanoate, ethyl decanoate, and ethyl dodecanoate were the major compounds detected. Esters are known to be produced by yeasts during fermentation and they affect the fruity flavors of alcoholic beverages such as wine and beer [14]. The levels of ethyl octanoate and ethyl decanoate in MSJ were much higher than those in the other samples. Ethyl octanoate is well known to show wine, brandy, and fruity aromas; ethyl dodecanoate has an oily brandy aroma and is an important flavor component in a distilled liquor.

Alcohols constitute a group of compounds with the highest concentration in distilled beverages. The level of these compounds depends on the raw material, fermentation conditions, and distillation technique [21]. Among the 10 alcohol compounds identified, isobutyl alcohol (sweet and musty aroma), 1-hexanol (mild, sweet, green aroma), 3, 7-dimethyl-6-octenol (rose-like aroma), and 2-phenylethanol (rose, honey-like aroma) were detected in more than 7 samples. Acids were considered as important contributors to flavor. Octanoic acid (unpleasant flavor) was detected in eight samples and decanoic acid (unpleasant flavor) was detected in four samples. Acid is an important component because even a small amount in combination with alcohol will form esters [22]. The samples made with atmospheric distillation, such as MSJ, showed much higher levels of these acids than those of the other samples. As also reported in volatile compounds of spirits by different distillations and filtrations, the sample with the atmospheric distillation showed higher levels of esters and alcohols compared to other samples with vacuum distillation [23].

**Figure 1:**
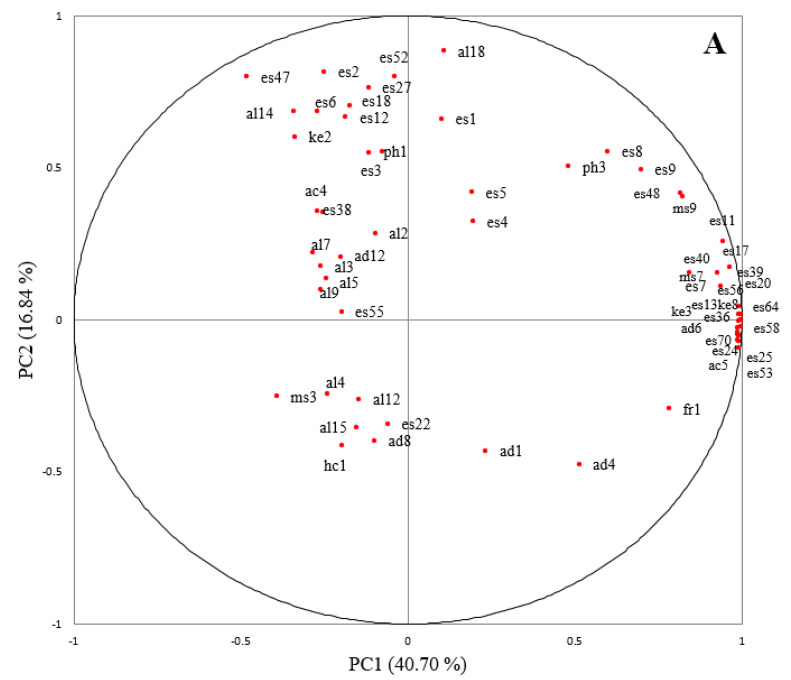

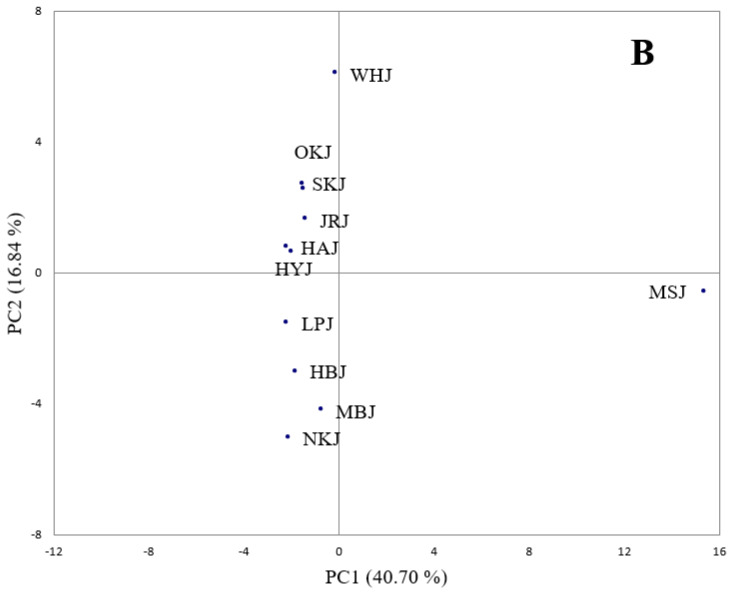
Principal component analysis loadings for 59 volatile compounds (**A**) and scores for the 11 distilled *soju* samples (**B**). The sample and volatile compound codes are defined in Table 1, Table 2 and Table 3.

To determine the overall distributions of the volatiles with the separation of samples, a principal component analysis was performed using the 59 volatile compounds detected more than three samples. The first principal component (PC1) showed an explanatory power of 40.70%, while the second principal component (PC2) showed an explanatory power of 16.84% as shown in Figure 1A,B. Figure 1A shows a plot of volatile compounds and Figure 1B depicts the distribution of each sample. Across the PC1, MSJ showed a prominent contrast to other remaining samples. Concentrations of various esters such as ethyl octanoate (es17), ethyl decanoate (es36), ethyl dodecanoate (es53), and ethyl tetradecanoate (es64) were particularly high. A large amount of longer chain esters was detected in MSJ using an atmospheric distillation, whereas a small amount of those compounds was detected in samples using vacuum distillation. Moreover, across the PC2, there was a distinction among samples based on major ingredients used for brewing. Samples WHJ and SKJ, which used only barley for brewing, showed higher concentrations of esters such as isoamyl acetate (es6), ethyl octanoate (es17), and phenethyl acetate (es52), while NKJ and LPJ, which used only rice, showed generally lower concentrations of most volatile compounds. This ultimately could be explained as a difference in the composition of volatile compounds based on distillation methods and raw materials.

### 3.2. Sensory Characteristics of Distilled Soju Samples by Descriptive Analysis

Table 4 outlines the mean intensities of sensory attributes obtained for the 11 samples along with their significance testing. WHJ and SKJ made from barley showed higher intensities in barely_A and yeast_T. Raw materials used for the production and distillation methods were one of the most important elements influencing the flavor characteristics of distilled liquors [11]. Not surprisingly, JRJ and OKJ samples blended with spirits matured in oak barrels scored higher in the brandy (oak-like) aroma compared to those in other samples. The maturing of alcoholic beverages in wooden barrels after distillation has been known to enhance their quality through additional aroma development because of interaction with the wooden barrels [13,24]. The intensity of the bitter taste was the highest in the MSJ sample, manufactured by atmospheric distillation. This was consistent with a previous study that showed that unlike other vacuum distillation methods, atmospheric distillation yielded distilled *soju* with strong alcohol-related flavors and tastes [11]. Samples with higher alcohol levels like MSJ, HBJ, HAJ, and WHJ might be rated with lower intensities compared to the original samples. However, the purpose of the sensory descriptive analysis in this study was to find out important sensory attributes in the selected sample set and to compare those among samples rather than analyze each sample thoroughly.

**Figure 2:**
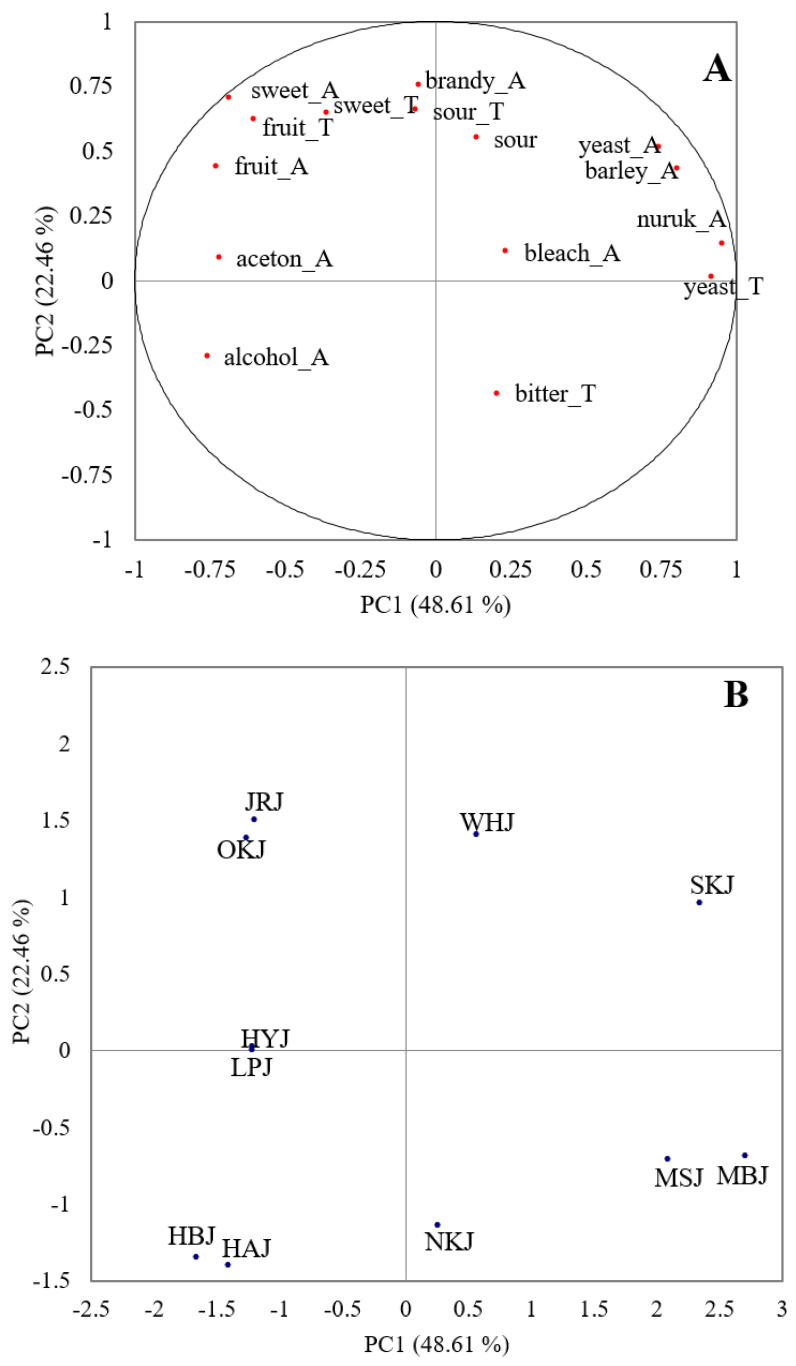
Principal component analysis loadings for 22 sensory attributes (**A**) and scores for the 11 distilled *soju* samples (**B**). The samples and sensory attribute codes are defined in Table 1 and Table 2. PC1, first principal component; PC2, second principal component.

The overall sensory configuration of these samples was two-dimensional, as shown in Figure 2, which summarizes the results from the PCA. Alcohol taste and overall texture/mouthfeel related sensory attributes were excluded from the analysis because they did not show significant differences in 3-factor mixed model ANOVA (*p* > 0.05). As shown in Figure 2, PC1, and PC2 account for 48.61% and 22.46% of the data deviation, respectively. PC1 was a contrast between aromas and flavor/tastes related to yeast, barely, and *nuruk* versus those with fruit-related or acetone aroma. Fruit_A showed a significant positive correlation with sweet_A (*r =* 0.815) and fruit_T (*r =* 0.723) (*p <* 0.05). Alcohol_A displayed a positive correlation with aceton_A (*r =* 0.64). PC2 also showed a distinctive contrast between bitterness vs. brandy aroma and sourness. The distribution of samples on this plot (Figure 2B) was related to the mean intensities of the investigated samples (Table 4). OKJ and JRJ were located to the far left along the PC1, indicating high levels in sweet_T, fruits_A, and brandy_A with sourness. On the opposite side of PC1, MBJ and MSJ were located in the same direction with bitter_T and yeast/*nuruk* related attributes in which those attributes showed higher intensities among the tested samples.

Mainly by PC2, WHJ and SKJ were located in the first quadrant with higher levels of aroma and taste attributes related to yeast and *nuruk*, also with barley aroma. WHJ and SKJ samples were made with 100% barely; therefore, their high scores were assumed to be related to the major ingredient. In contrast, HBJ, HAJ, and NKJ, produced mainly from rice using vacuum distillation, showed weak aroma in general sensory characteristics. In addition, sensory characteristics were found to be weak in traditionally distilled liquors using 100% non-glutinous rice compared to those using brown rice, barley, or glutinous rice as also reported in Kim et al. [16].

### 3.3. Relationships between Sensory Characteristics and Volatile Compounds

From the correlation analysis, fruit_A was significantly correlated with isobutyl acetate (es2; *r* = 0.73) and ethyl butyrate (es3; *r* = 0.64) (*p* < 0.05). Sweet_A also displayed a significant positive correlation with isobutyl acetate (es2; *r* = 0.63). All the sweet and fruit-related sensory attributes were negatively correlated with aldehydes such as hexanal (ad1), nonanal (ad4), and 2-pentyl furan (fr1) (*p* < 0.05). Ethyl nonanoate (*r* = 0.63), ethyl lactate (*r* = 0.64), ethyl tetradecanoate (*r* = 0.64), and ethyl hexadecanoate (*r* = 0.64) displayed positive correlations with bleach_A (*p* < 0.05), which was a dominant aroma character of MSJ. These compounds exhibited fatty and waxy odor [20]. This appeared to be consistent with these esters having an aroma that is unique to atmospheric distilled products compared to vacuum distilled samples [23]. The 2-methoxy-phenol (ph1) showed a significantly positive correlation with barley_A (*r* = 0.68) and yeast_A (*r* = 0.86) (*p* < 0.01). Nuruk_A was significantly correlated with dihydro-5-pentyl-2(3H)-Furanone (ke8; *r* = 0.62) and 2-pentyl furan (fr1; *r* = 0.73) (*p* < 0.05).

**Figure 3:**
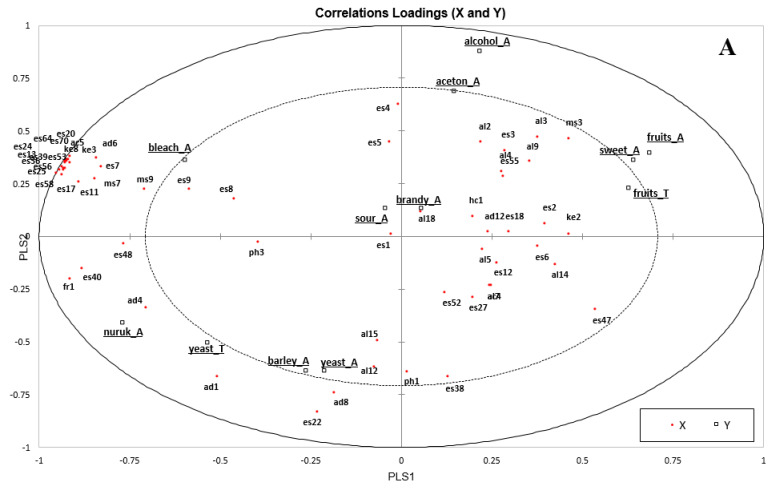

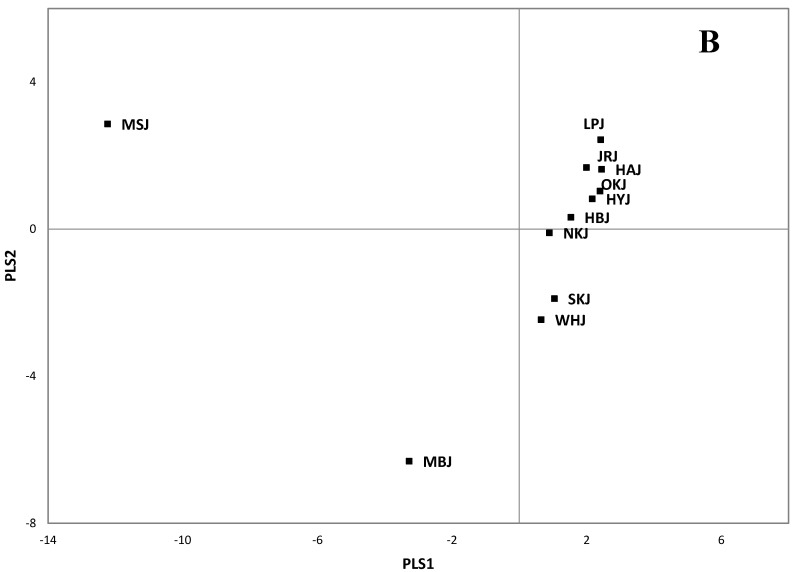
Correlation loadings (**A**) from PLS of 59 volatile compounds (X) and 12 sensory attributes (Y), and samples scores (**B**) of 11 commercially distilled *soju* samples (closed squares). Explained variance for X (GC data) is 37.9% and 13.1% for PC1 and PC2, respectively, and for Y (Sensory data) is 22.4% and 24.7%, respectively. The sample and volatile compound codes are defined in Table 1, Table 2 and Table 3.

Partial least square regression analysis (PLSR) was widely applied to elucidate relationships between instrumental and sensory data in a multivariate way, rather that examining one-to-one relationships between volatiles and sensory data [25,26]. The first and second PLS components were calculated by cross-validation based on 59 volatile data points (*X* variable) and twelve aroma attributes (*Y* variable) excluding tastes of sweet, sour, bitter, and mouth-feel attributes. The two PLS components explained 47% of the total variance in the sensory data and 51% of the GC data. Figure 3A shows the relationship between volatile compounds and sensory attributes, while Figure 3B depicts the distribution of each sample. The center ellipsoid in Figure 3A indicates 50% of the explained variation. Many volatile compounds were located inside the ellipsoid, which means they did not contribute to the model considerably. However, the casual relationship between GC and sensory data could be explained in this model.

Similar to the results of the PCA for sensory data, fruit and sweet related attributes were clustered together in the positive side of PLS1, while *nuruk*, barley, and yeast-related attributes were located in the negative side of PLS1. Esters like ethyl lactate (es13), ethyl nonanoate (es24), ethyl tetradecanoate (es64), and ethyl hexadecanoate (es70) were mainly distributed in the far outer negative direction of PLS1, displaying a strong association with bleach_A. Along the positive side of PLS2, the same directions for alcohol_A and aceton_A are shown with volatile compounds such as isobutyl alcohol (al2), 1-butanol (al3), 1-pentanol (al4), and benzoyl bromide (ms3). In contrast, in the negative side of PLS2, barley_A, yeast_A, and yeast_T were closely positioned with hexanal (ad1), nonanal (ad4), benzaldehyde (ad8), and 2-methoxy-phenol (Ph1). These volatile compounds are known to have fatty-green, grassy, and almond odors [27].

Similar to the results of the PCA for sensory and volatile data, samples were mainly separated by their distillation methods. In Figure 3B, MSJ with larger amounts of longer chain esters was positioned in the far negative side of PLS1. JRJ, OKJ, and HYJ were closely located with sensory attributes like sweet_A, fruits_A, and fruit_T on the positive direction of PLS1. The vacuum distillation method that separates compounds in low temperature shows the advantages of having less heated/burnt aroma and maintaining various fruity and sweet aroma compounds well.

## 4. Conclusions

The differences in volatile compositions and sensory characteristics in 11 commercially distilled *soju* samples were investigated. PCA on the volatile compounds showed the main difference according to the distillation method. MSJ produced by atmospheric distillation had a large amount of longer chain esters, showing a contrast with the other samples produced by vacuum distillation. There were differences in sensory characteristics based on the raw material, distillation, and aging method. PLSR analysis using volatile compounds and sensory attributes was applied to see any relationship between these two data sets. In terms of the positive side of PLS1, products mainly brewed using rice by vacuum distillation were variously dispersed. Using barley or other cereals than rice as a main ingredient, MBJ, WHJ, and SKJ showed associations with barley or yeast-related aromas. Main ingredients, distillation, and aging methods greatly affect the volatile and sensory characteristics of distilled *soju* samples. Those characteristics can critically influence consumer preference. Based on these findings, this study aims to contribute to the development of new products through consumer preference studies in the future.

## Figures and Tables

**Table 1:** Information about the 11 commercially distilled *soju* samples.

Code	Alcohol content (%, W/V)	Distillation Method	Raw Materials	Aging Method
MSJ	45	Atmospheric Distillation	non-glutinous rice, glutinous rice	
HBJ	35	Vacuum Distillation	rice, barley	
HAJ	35	Vacuum Distillation	rice	
SKJ	23	Vacuum Distillation	barley	
WHJ	35	Vacuum Distillation	barley	
NKJ	19.8	Vacuum Distillation	rice	
LPJ	21	Vacuum Distillation	rice	
JRJ	25	Vacuum Distillation	rice	oak barrels
HYJ	25	Vacuum Distillation	rice	
OKJ	25	Vacuum Distillation	rice	Limousin oak
MBJ	23	Vacuum Distillation	rice, Italian millet, sorghum	

**Table 2:** Sensory code, attributes, definitions, and physical standards of distilled *soju.*

Attribute	Code	Written Definition	Physical Standards
***Aroma***			
Alcohol	alcohol_A	The smell associated with alcohol	25% (W/V) ethanol
Sour	sour_A	Sour aroma	1 mL vinegar/ 50 mL distilled water
Sweet	sweet_A	The smell associated with honey aroma	Honey 20 g/100 mL distilled water
Fruits	fruits_A	From fruit aroma (ex: pear)	Crushed pear 30 g/30 mL distilled water
Nuruk	nuruk_A	Nuruk aroma	Nuruk 10 g/100 mL distilled warm water
Yeast	yeast_A	From activated yeast aroma	Yeast 0.1% in 10% warm sugar solution overnight
Brandy	brandy_A	Brandy aroma	70 mL brandy/30 mL distilled water
Acetone	aceton_A	Acetone aroma	5 mL acetone/100 mL distilled water
Bleach	bleach_A	From Bleach aroma in hydrogen peroxide	2 mL hydrogen peroxide/100 mL distilled water
Barley	barley_A	From a typical barely	a typical barely drink
***Flavor/taste***			
Alcohol	alcohol_T	Alcohol taste	25% (W/V) ethanol
Sweet	sweet_T	Sweet taste	sucrose 6% (W/V)
Fruits	fruits_T	From fruits taste (ex: green grape)	50 mL white wine/500 mL distilled water
Sour	sour_T	Sour taste	Citric acid 0.25% (W/V)
Bitter	bitter_T	Bitter taste	Anhydride caffeine 0.1%
Yeast	yeast_T	From activated yeast taste	Yeast 0.1% in 10% warm sugar solution overnight
***Texture/Mouthfeel***			
Astringent	astrin	Mouthfeel of dryness	Aluminum sulfate 0.1% (W/V)
Body	body	Full-bodyness while tasting	No physical standards
Continuation	contin	The feeling of continuing taste	No physical standards
Pungent	pung	Pungent taste	No physical standards
Swallow	swall	Irritation while swallowing	No physical standards
Cooling sensation	cool	Cool feeling	Crushed peppermint candy

**Table 3:** Volatile components (mg/L) ^1^ in the commercially distilled *soju* samples.

No.	Code	RI ^2^	Compound	MSJ	WHJ	JRJ	HBJ	SKJ	HAJ	MBJ	HYJ	NKJ	LPJ	OKJ	Id ^3^
1	es1	945	ethyl acetate	3.71	4.34	6.92	-	4.4	3.6	3.5	4.37	-	2.7	3.57	A
2	es2	1006	Isobutyl acetate	0.05	0.38	0.17	0.03	0.14	0.29	0.05	0.35	-	0.09	0.34	A
3	es3	1033	ethyl butyrate	0.44	0.4	0.63	0.11	0.68	0.71	0.25	0.85	-	0.87	0.8	B
4	es4	1047	ethyl 2-methylbutanoate	0.24	0.1	0.34	0.07	0.15	0.2	-	-	-	0.36	0.22	A
5	es5	1063	ethyl 3-methylbutanoate	0.16	0.05	0.33	0.02	0.15	0.07	-	-	-	0.13	0.19	A
6	es6	1111	Isoamyl acetate	1.38	29.4	5.13	2.67	15.27	8.16	2.11	31.28	0.41	14.41	13.36	A
7	es7	1123	ethyl valerate	0.94	0.08	0.33	0.06	0.13	-	0.04	-	-	-	0.2	A
8	es8	1210	Isoamylformate	38.08	34.45	10.74	-	-	-	-	-	-	-	36.23	A
9	es9	1219	ethyl hexanoate	23.72	14.29	10.74	2.97	14.11	7.09	8.67	14.16	0.44	13.61	11.07	A
10	es11	1318	ethyl heptanoate	4.49	1.38	0.72	0.3	1.12	0.46	0.51	1.35	-	0.28	1.04	A
11	es12	1337	Isobutyl hexanoate	-	0.26	-	-	0.18	0.3	-	-	-	-	0.11	A
12	es13	1337	ethyl lactate	14.39	-	-	-	0.09	0.15	0.51	-	-	-	-	A
13	es17	1429	ethyl octanoate	238.85	70.97	15.73	15.48	26.79	49.81	33.9	42.57	0.5	6.34	46.25	B
14	es18	1438	ethyl (2E)-2-heptenoate	-	0.44	0.4	-	-	-	-	0.3	-	-	0.27	B
15	es20	1444	Isopentyloctanoate	5.05	0.59	0.12	0.08	0.18	0.32	-	0.21	-	-	0.22	A
16	es22	1471	ethyl 7-octanoate	-	0.38	-	-	0.12	-	3.28	0.19	-	-	-	A
17	es24	1520	ethyl nonanoate	17.88	1.21	0.1	0.17	0.13	0.39	-	0.61	-	-	0.45	B
18	es25	1528	Ethyl dl-2-hydroxycaproate	2.09	0.09	0.08	-	0.08	0.13	0.2	-	-	0.2	-	B
19	es27	1538	EthylE-2-octenoate	-	1.23	-	0.11	0.35	-	-	0.38	-	-	0.45	A
20	es36	1625	ethyldecanoate	524.73	29.74	0.62	3.1	0.65	8.13	2.23	4.07	0.07	0.24	4.33	A
21	es38	1655	ethyl benzoate	-	0.18	0.05	0.03	0.07	0.08	0.15	0.13	-	0.02	-	A
22	es39	1657	ethyl trans-4-decenoate	10.65	1.22	-	-	0.11	-	-	-	-	-	-	B
23	es40	1664	Diethyl butanedioate	18.44	10.49	0.66	0.14	3.61	4.55	8.74	0.5	0.14	0.78	0.7	B
24	es47	1764	methyl 2-hydroxy-benzoate	-	0.65	0.27	0.23	0.46	0.35	0.25	0.33	0.08	0.23	0.45	B
25	es48	1771	Ethyl benzeneacetate	1.54	1.12	0.15	0.04	0.62	-	0.3	0.2	-	0.1	0.23	A
26	es52	1799	phenethyl acetate	3.86	29.23	1.16	1.31	11.55	1.2	1.89	12.93	0.16	1.73	12.96	A
27	es53	1833	ethyl dodecanoate	160.25	1.07	0.27	0.16	0.26	1.45	0.79	0.68	-	0.19	0.4	B
28	es55	1854	neopentylnonyl oxalate	-	-	-	-	-	-	-	-	0.04	0.09	0.14	B
29	es56	1866	ethyl3-phenylpropanoate	2.02	-	-	-	-	0.03	0.17	-	-	-	-	B
30	es58	1880	ethyl3-methylbutyl butanediate	4.92	0.65	0.03	-	0.14	0.46	0.31	-	-	-	0.05	A
31	es64	2031	ethyltetradecanoate	69.27	0.48	0.13	0.17	0.16	0.82	0.34	0.78	-	-	0.36	A
32	es70	2228	ethyl hexadecanoate	20.66	0.34	0.06	0.12	0.08	0.22	0.05	1.72	-	-	0.2	A
33	al2	1098	Isobutyl alcohol	0.8	0.65	1.29	0.15	0.48	2.53	0.47	0.98	0.47	1.04	1.08	A
34	al3	1146	1-Butanol	-	-	0.22	-	-	0.26	-	-	-	0.15	0.09	A
35	al4	1202	1-pentanol	-	-	-	-	-	-	-	28.05	13.58	33.49	-	A
36	al5	1204	Isoamyl alcohol	-	-	42.09	7.58	30.44	41.83	17.75	-	-	-	-	A
37	al7	1341	1-hexanol	-	0.14	0.29	-	0.12	-	0.14	-	0.09	0.13	0.1	A
38	al9	1474	2-ethyl-1-hexanol	-	-	0.2	0.17	-	0.68	-	-	0.07	0.06	0.28	A
39	al12	1641	1-nonanol	-	-	-	-	-	-	0.84	0.33	0.06	-	0.45	B
40	al14	1745	3,7-dimethyl-6-Octenol	-	0.18	0.04	0.03	0.13	0.2	0.07	0.17	-	0.02	0.23	B
41	al15	1763	2-Methyl-2-nonanol	-	-	-	-	-	0.11	0.13	-	0.02	-	-	A
42	al18	1887	2-phenylethanol	14.5	19.95	18.02	3.15	24.52	17.28	4.3	8.93	1.6	7.54	21.28	A
43	ad1	1076	hexanal	0.26	-	-	-	-	0.04	0.89	-	-	-	-	A
44	ad4	1379	nonanal	0.6	0.15	-	-	-	0.3	0.7	-	0.44	0.06	0.15	A
45	ad6	1456	2-furancarboxal dehyde (furfural)	8.17	-	0.36	-	-	-	-	-	-	-	0.75	A
46	ad8	1514	benzaldehyde	-	-	0.09	-	-	0.09	0.85	-	0.06	-	0.14	B
47	ad12	1640	4-methyl-benzaldehyde	-	-	0.24	0.09	0.31	-	-	-	-	0.09	-	A
48	ac4	2034	octanoic acid	-	0.18	0.47	0.02	0.24	0.33	0.4	-	-	0.03	0.62	B
49	ac5	2245	Decanoic acid	9.03	-	-	-	-	0.31	0.11	-	-	-	0.31	A
50	ke2	1376	2-nonanone	-	0.15	0.06	0.13	0.11	0.14	-	-	-	0.05	0.11	B
51	ke3	1582	2-undecanone	1.61	0.15	-	-	-	0.07	0.06	-	-	-	0.08	B
52	ke8	2010	dihydro-5-pentyl-2(3H)-Furanone	0.57	0.04	-	-	0.03	0.03	0.02	-	-	-	0.02	B
53	ms3	1270	Benzoylbromide	-	-	0.09	0.09	-	0.18	-	0.25	0.13	0.2	-	B
54	ms7	1407	1,3-bis(1,1-dimethylethyl)-Benzene	1.68	0.72	0.37	0.54	0.2	0.5	0.22	0.31	0.15	0.19	0.27	B
55	ms9	1411	1,2,3,5-tetramethyl-benzene	0.79	0.49	0.39	0.07	0.1	0.09	0.04	-	-	-	0.13	B
56	ph1	1841	2-methoxy-Phenol	-	0.44	-	-	0.31	-	0.15	-	-	-	-	B
57	ph3	2281	2,4-bis(1,1-dimethylethyl)-Phenol	0.91	0.88	0.74	0.86	0.79	0.51	0.55	0.66	0.35	0.37	0.62	A
58	hc1	945	1,1-diethoxy-ethane	-	-	0.69	3.51	-	-	-	-	1.32	-	0.53	A
59	fr1	1222	2-pentyl-furan	1.06	-	0.08	-	-	-	0.85	-	-	-	0.13	A

-; Not detected. ^1^ Average of the mg/L (*w*/*v*) = (Area of each compound × Amount of internal standard)/(Area of internal standard × Amount of sample/10^6^). ^2^ Kovats indices of unknown compounds on Stabilwax column. ^3^ Volatiles were identified based on the following criteria: (A) mass spectrum and retention index consistent with previous reports, or an authentic standard or (B) mass spectrum consistent with that of the Wiley 275 mass spectrum database.

**Table 4:** Mean sensory intensity ratings for eleven distilled *soju* samples determined by descriptive analysis from a panel of ten judges over duplicate replications.

Sensory Code ^A^	Sample Code ^B^											
	JRJ	HYJ	LPJ	MBJ	NKJ	WHJ	SKJ	OKJ	HAJ	MSJ	HBJ	LSD (5%)
**Aroma**												
alcohol_A	5.95 ^abc^	6.45 ^a^	6.25 ^ab^	4.75 ^d^	6.20 ^ab^	5.50 ^bcd^	5.20 ^cd^	5.95 ^abc^	6.50 ^a^	6.05 ^ab^	6.05 ^ab^	0.76 ***
sour_A	4.70 ^bcd^	4.95 ^abc^	5.15 ^abc^	4.45 ^cd^	5.20 ^ab^	5.50 ^a^	4.95 ^abc^	5.25 ^ab^	4.45 ^cd^	5.05 ^abc^	4.15 ^d^	1.01 **
sweet_A	6.85 ^a^	6.20 ^abc^	6.15 ^abcd^	4.75 ^f^	5.40 ^def^	6.40 ^ab^	5.40 ^def^	6.60 ^a^	5.65 ^bcde^	4.85 ^f^	5.50 ^cdef^	0.79 ***
fruit_A	5.60 ^bcd^	6.70 ^a^	6.20 ^abc^	4.35 ^g^	5.10 ^defg^	6.05 ^abc^	5.40 ^cdef^	6.40 ^ab^	5.65 ^bcd^	4.60 ^fg^	5.55 ^cde^	1.04 ***
yeast_A	4.35 ^ef^	4.75 ^cde^	4.50 ^def^	5.55 ^abc^	4.05 ^ef^	5.85 ^ab^	6.15 ^a^	4.50 ^def^	3.90 ^f^	4.80 ^cde^	3.80 ^f^	1.07 ***
nuruk_A	3.95 ^bcd^	3.50 ^cde^	3.70 ^cde^	5.50 ^a^	4.00 ^bc^	4.65 ^b^	5.50 ^a^	3.65 ^cde^	3.20 ^de^	6.05 ^a^	3.10 ^e^	0.96 ***
barely_A	4.30 ^cd^	4.25 ^cd^	4.15 ^cde^	5.20 ^ab^	4.90 ^abc^	5.10 ^ab^	5.60 ^a^	4.65 ^bc^	3.45 ^e^	4.65 ^bc^	3.85 ^de^	0.79 ***
aceton_A	5.90 ^a^	5.85 ^a^	5.60 ^ab^	4.70 ^cd^	4.50 ^d^	4.80 ^bcd^	4.45 ^d^	5.45 ^abc^	5.45 ^abc^	5.35 ^abc^	5.40 ^abc^	1.03 ***
bleach_A	5.15 ^abc^	4.65 ^bcd^	5.25 ^ab^	4.60 ^bcd^	4.55 ^bcd^	4.45 ^cd^	4.25 ^d^	4.20 ^d^	4.05 ^d^	5.65 ^a^	3.95 ^d^	1.04 ***
brandy_A	6.70 ^a^	4.50 ^cde^	4.35 ^cde^	4.70 ^bcd^	4.25 ^de^	4.80 ^bcd^	5.00 ^bc^	6.00 ^a^	4.50 ^cde^	4.85 ^bcd^	3.85 ^e^	1.14 ***
**Flavor/Taste**												
alcohol_T	6.20	6.50	6.40	6.05	6.25	6.00	5.85	6.00	6.85	6.15	6.80	NS
sweet_T	6.15 ^ab^	5.60 ^bcd^	6.10 ^ab^	5.35 ^cd^	5.30 ^d^	6.05 ^abc^	6.30 ^ab^	6.65 ^a^	5.70 ^bcd^	5.60 ^bcd^	6.20 ^ab^	0.90 **
fruit_T	5.45 ^ab^	5.00 ^abc^	5.10 ^abc^	4.10 ^e^	4.75 ^bcde^	5.60 ^a^	4.90 ^abcd^	5.35 ^ab^	4.55 ^cde^	4.25 ^de^	5.35 ^ab^	0.86 **
sour_T	4.90 ^abc^	5.00 ^ab^	5.10 ^ab^	4.25 ^cd^	4.45 ^bcd^	4.90 ^abc^	5.15 ^a^	4.80 ^abc^	4.10 ^d^	4.75 ^abcd^	4.70 ^abcd^	0.73 *
bitter_T	4.95 ^cd^	5.95 ^ab^	5.25 ^bcd^	5.30 ^bcd^	5.65 ^abc^	4.75 ^d^	5.50 ^abc^	5.45 ^abcd^	5.35 ^bcd^	6.10 ^a^	5.30 ^bcd^	0.92 **
yeast_T	4.25 ^fg^	4.80 ^cdef^	4.15 ^fg^	5.55 ^ab^	5.00 ^bcde^	5.00 ^bcde^	5.90 ^a^	4.45 ^efg^	4.70 ^def^	5.50 ^abc^	3.90 ^g^	0.96 ***
**Texture/Mouthfeel**												
Pung	5.40	5.15	5.75	5.50	5.70	5.45	5.30	5.60	5.00	5.35	5.15	NS
Astrin	5.00	5.05	4.90	5.10	5.00	5.00	5.25	4.70	4.45	5.00	4.30	NS
Cool	5.80	5.45	5.95	5.35	5.30	5.50	5.35	5.75	5.80	5.35	5.70	NS
Contin	5.50	5.20	5.85	5.50	5.65	5.20	5.55	5.40	5.05	5.35	5.40	NS
Swall	5.75	5.45	5.70	5.95	5.70	5.15	5.15	5.50	5.50	5.10	5.35	NS
Body	5.25	5.00	4.90	5.35	5.05	4.50	5.00	5.05	4.85	5.10	4.60	NS

^a–g^ Mean values with the same letter in a row are not significantly different, with significance set at *p <* 0.05 by Fisher’s least significant difference test. NS = Not Significant, * = (*p* < 0.05), ** = (*p* < 0.01), *** = (*p* < 0.001). The intensity of the attributes ranged from 0 to 9 (0, none; 1, very weak, 5: moderate, 9: very strong). ^A,B^ The sensory attribute codes and samples are defined in Table 1 and Table 2.
